# The Acute Phase Protein Ceruloplasmin as a Non-Invasive Marker of Pseudopregnancy, Pregnancy, and Pregnancy Loss in the Giant Panda

**DOI:** 10.1371/journal.pone.0021159

**Published:** 2011-07-13

**Authors:** Erin L. Willis, David C. Kersey, Barbara S. Durrant, Andrew J. Kouba

**Affiliations:** 1 Department of Conservation and Research, Memphis Zoological Society, Memphis, Tennessee, United States of America; 2 Department of Reproductive Sciences, Center for Species Survival, Smithsonian Conservation Biology Institute, National Zoological Park, Front Royal, Virginia, United States of America; 3 Reproductive Physiology Division, Institute for Conservation Research, San Diego Zoo Global, Escondido, California, United States of America; Baylor College of Medicine, United States of America

## Abstract

After ovulation, non-pregnant female giant pandas experience pseudopregnancy. During pseudopregnancy, non-pregnant females exhibit physiological and behavioral changes similar to pregnancy. Monitoring hormonal patterns that are usually different in pregnant mammals are not effective at determining pregnancy status in many animals that undergo pseudopregnancy, including the giant panda. Therefore, a physiological test to distinguish between pregnancy and pseudopregnancy in pandas has eluded scientists for decades. We examined other potential markers of pregnancy and found that activity of the acute phase protein ceruloplasmin increases in urine of giant pandas in response to pregnancy. Results indicate that in term pregnancies, levels of active urinary ceruloplasmin were elevated the first week of pregnancy and remain elevated until 20–24 days prior to parturition, while no increase was observed during the luteal phase in known pseudopregnancies. Active ceruloplasmin also increased during ultrasound-confirmed lost pregnancies; however, the pattern was different compared to term pregnancies, particularly during the late luteal phase. In four out of the five additional reproductive cycles included in the current study where females were bred but no birth occurred, active ceruloplasmin in urine increased during the luteal phase. Similar to the known lost pregnancies, the temporal pattern of change in urinary ceruloplasmin during the luteal phase deviated from the term pregnancies suggesting that these cycles may have also been lost pregnancies. Among giant pandas in captivity, it has been presumed that there is a high rate of pregnancy loss and our results are the first to provide evidence supporting this notion.

## Introduction

With over 300 pandas in captivity around the world, substantial progress has been made in the ex-situ (captive) conservation of this species in recent years, but as with any small population, careful management is required to maintain genetic diversity and to prevent inbreeding [1]. Although significant progress has been made in growing the captive population, giant pandas remain a challenge to breed in captivity due to the single estrous period per year with sexual receptivity lasting only one to three days, mate incompatibility, lack of sexual interest, and failure to give birth following optimal timing of insemination. Female giant pandas spontaneously ovulate [2] and undergo a phenomenon known as pseudopregnancy if not pregnant, wherein a female's reproductive hormones are similar in concentration and length during the non-pregnant luteal phase as during pregnancy. This makes pregnancy determination impossible by diagnostic hormonal tests typically used in other mammals [2], [3]. In many other species, pregnancy can be diagnosed from hormonal monitoring of the luteal steroid, progesterone, or its excreted metabolites in urine and feces. However, progestagen patterns between pregnant and pseudopregnant pandas are indistinguishable [2], [3], [4], [5], [6]. Furthermore, these luteal phases are unpredictable in length. They consist of a variable primary phase lasting anywhere from around 60 to 122 days, which is characterized by a slight increase in progestagens above baseline levels [4], [5]. The primary rise of progestagens is then followed by a more consistent secondary phase which comprises a substantial increase in progestagens above baseline lasting 40–50 days [4], [5]. In a pregnant giant panda, the embryo remains quiescent in embryonic diapause until the secondary rise of progestagens when implantation is suspected to occur and the fetus begins to grow rapidly [2], [3], [7], [8], [9].

Pregnant females do not show marked changes in behaviors until the last two weeks of gestation, but these behaviors are often inconsistent among females and pseudopregnant females may also exhibit similar changes in behavior prior to the end of the luteal phase [4]. In a limited number of cases, ultrasonography has been helpful in the detection of a fetus or gestational sac but is only applicable when used late in pregnancy at about 2 to 3 weeks prior to parturition, following the delayed implantation [7], [8]. Even then, fetal detection can be challenging and requires the skill of an expert ultrasonographer as well as the cooperation of the animal. While ultrasonography has provided some evidence of failed pregnancies due to embryonic loss in giant pandas [4], [8], [10], if no indications of pregnancy are observed by ultrasound and a cub is not born after progestagens return to baseline, it is usually assumed the female was not pregnant. However, because ultrasound can only detect a fetus in late gestation and females are often uncooperative at this time, many lost pregnancies could very easily go undetected. During one term pregnancy, thermal imaging also showed promise for detecting growing fetal tissue and determining litter size in giant pandas at an earlier stage than ultrasound [11]. However, this technique still does not differentiate pseudopregnancy from pregnancy until after the time of implantation. For decades, researchers all over the world have been searching for a ‘magic bullet’ that would provide a pregnancy test for giant pandas and other exotic wildlife that undergo pseudopregnancy. To date, all tests utilizing steroid hormones and their metabolites, as well as other hormones such as relaxin, have proven ineffective for distinguishing between pregnancy and pseudopregnancy [2], [3], [5], [12]. Thus, the lack of a reliable pregnancy test continues to limit our ability to understand aspects of conception, delayed implantation/embryonic diapause, embryonic loss and pregnancy maintenance for this species.

Recently, our laboratory began to investigate alternative potential markers of pregnancy in the giant panda. Serum levels of some acute phase proteins typically associated with the immune system and inflammation were found to increase during pregnancy in mammals such as humans and dogs [13], [14], [15], [16]. However, daily and even weekly blood collections from endangered/exotic species are challenging to obtain. Therefore, we examined the possibility of whether a marker of inflammation, the acute phase protein ceruloplasmin, could be measured in urine collected non-invasively and also whether it could be used to distinguish between pregnancy and pseudopregnancy in the giant panda. Using this approach, we discovered that the activity of ceruloplasmin increased in urine of pregnant giant pandas compared to non-pregnant animals and that this increase occurs early in gestation allowing for a diagnosis of pregnancy within one week after breeding. Furthermore, the ceruloplasmin early pregnancy assay (CEP assay) indicated a very high level of pregnancy loss in captive pandas.

## Results

### Active urinary ceruloplasmin in known pseudopregnancies (no breeding) and term pregnancies

**Figure 1:**
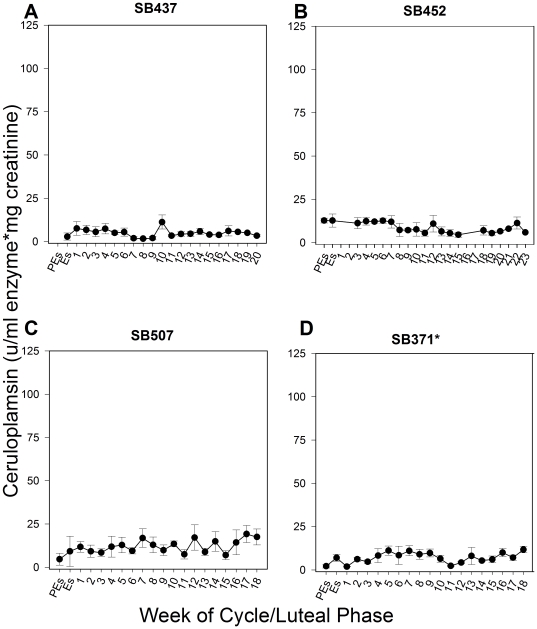
The levels of active ceruloplasmin in urine for female giant pandas during known pseudopregnant cycles when no breeding occurred. One known pseudopregnancy when no breeding or AI occurred is depicted for each female giant panda housed in U.S. institutions. Panel A: Known pseudopregnancy in 2002 for SB437 (SNZP). Panel B: Known pseudopregnancy in 2003 for SB452 (ZA). Panel C: Known pseudopregnancy in 2005 for SB507 (MZS). Panel D: Known pseudopregnancy in 1997 for SB371 (SDZ). Urinary progestagens were monitored in each cycle to ensure a normal luteal phase. Urine samples from every week of the reproductive cycle could not be obtained for SB452 during her known pseudopregnancy in 2003. PEs = Proestrus; Es = Estrus; Numerical numbers = weeks of the luteal phase. Variations in the x axis exist to accommodate variable concentrations and durations. Data are weekly means ± SEM; n = 4 reproductive cycles. *Samples from cycles obtained from SB371 were analyzed blind, without prior knowledge of cycle outcome.

**Figure 2:**
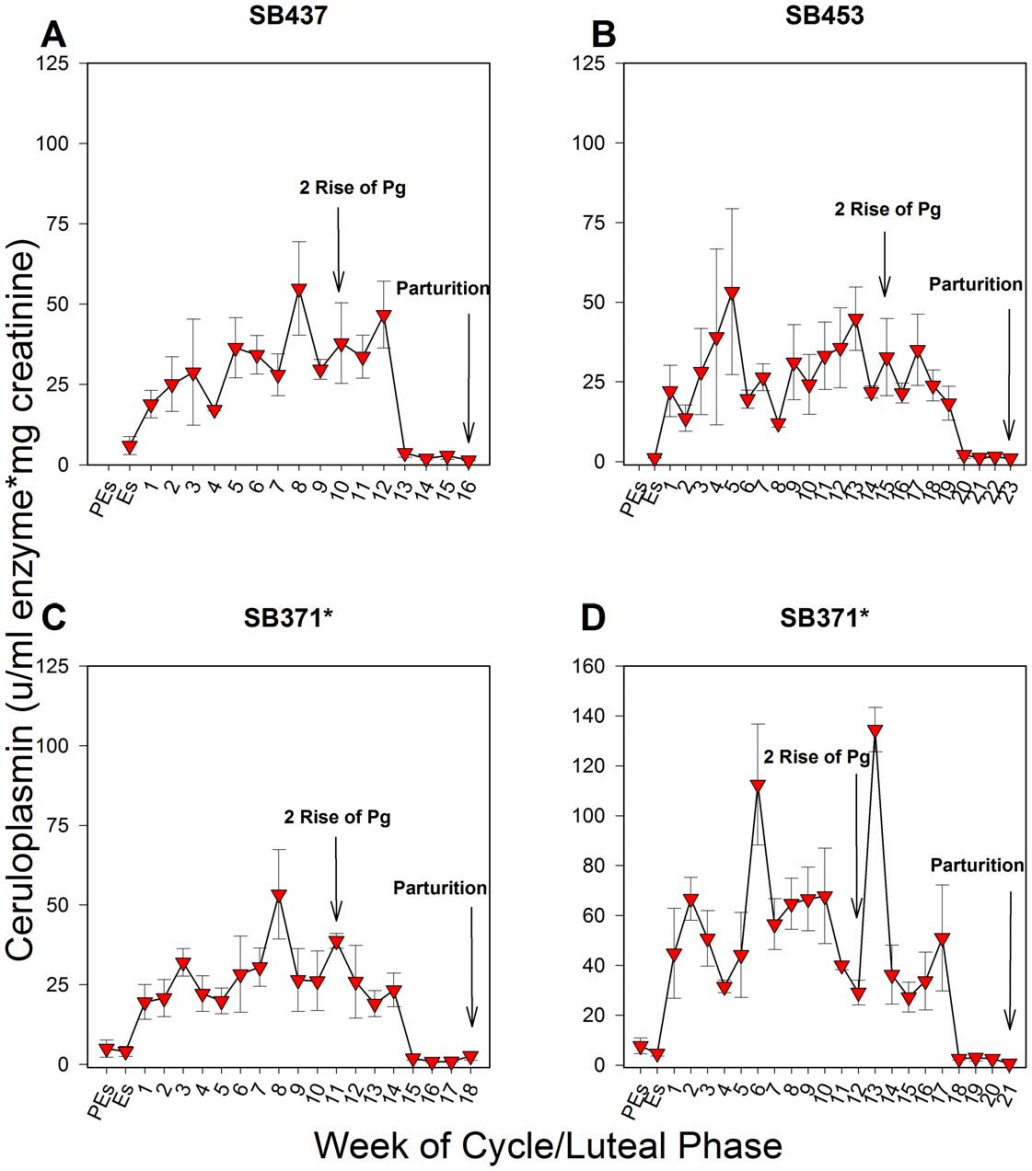
The levels of active ceruloplasmin in urine for female giant pandas during representative term pregnant cycles. Panel A: Term pregnancy in 2005 for SB437 (SNZP). Panel B: Term pregnancy in 2006 for SB452 (ZA). Panel C: Term pregnancy in 1999 for SB371 (SDZ). Panel D: Term pregnancy in 2003 for SB371 (SDZ). Animals were artificially inseminated (SB437; SB452) or artificially inseminated (SB371, reproductive cycle 1999) and bred naturally (SB371, reproductive cycle 2003). The amount of time active ceruloplasmin levels remained elevated in urine was dependent upon the gestation length of each term pregnancy, but levels consistently remained high through approximately half of the secondary rise in progestagens and then decreased to at or below proestrus/estrus levels 20–24 days prior to parturition in each pregnancy examined. SB371 was confirmed pregnant with twins by ultrasound in 2003, but only gave birth to one cub. All other term pregnant cycles characterized produced a singleton cub. Pg = Progestagen; PEs = Proestrus; Es = Estrus; Numerical numbers = weeks of the luteal phase. Variations in the x and y axes exist to accommodate variable concentrations and durations. Data are weekly means ± SEM; n = 5 reproductive cycles, representative cycles (n = 4) are presented herein. *Samples from SB371 were analyzed blind, without prior knowledge of cycle outcome.

**Figure 3 pone-0021159-g003:**
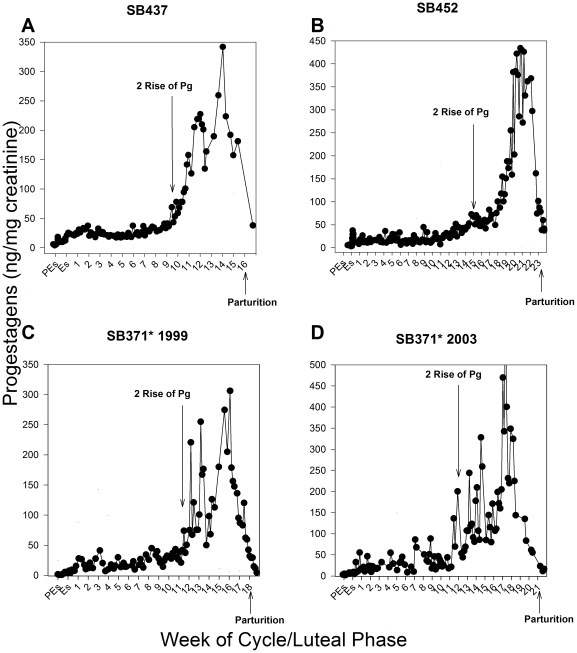
Urinary progestagens in representative pregnancies carried to term in female giant pandas. Black circles represent urinary progestagen concentrations; Pg = Progestagen; PEs = Proestrus; Es = Estrus; Numerical numbers = weeks of the luteal phase. Variations in the x and y axes exist to accommodate variable concentrations and durations. *Because urinary progestagen concentrations for SB371 were analyzed using a different antibody, caution must be taken when directly comparing progestagen data between SB371 and the other animals. However, the same trends were observed in the relationship between active ceruloplasmin and progestagens in urine using both antibodies.

No change in the levels of active urinary ceruloplasmin was observed throughout the estrous cycle in known pseudopregnant giant pandas that were not bred (Figure 1). However, in pregnancies carried to term, the levels of active ceruloplasmin in urine were elevated by 3.2- to 19.8-fold at week one of the luteal phase compared to the levels observed at proestrus/estrus (P≤0.05; Figure 2). These levels then remained high, ranging from 2.8- to 17.5-fold above proestrus/estrus levels, until 20–24 days prior to birth (P≤0.05; Figure 2). The secondary rise in progestagens was calculated for each luteal phase in cycles where breeding or artificial insemination (AI) occurred to determine the relationship between the pattern of ceruloplasmin and progestagens. In term pregnancies, ceruloplasmin remained increased for approximately half of the secondary rise in progestagens (Figure 3), ranging from 42–71% of the secondary progestagen rise, and then declined late in the luteal phase to at or below proestrus/estrus levels for the remainder of the cycle (Figures 2, 3). Cycles obtained from SB371 (San Diego Zoo; SDZ) were analyzed without prior knowledge of cycle outcome. The CEP assay accurately determined the known pseudopregnancy cycle and both term pregnancy cycles from SB371 included in this study.

### Active urinary ceruloplasmin in confirmed lost pregnancies and suspected lost pregnancies after breeding

**Figure 4 pone-0021159-g004:**
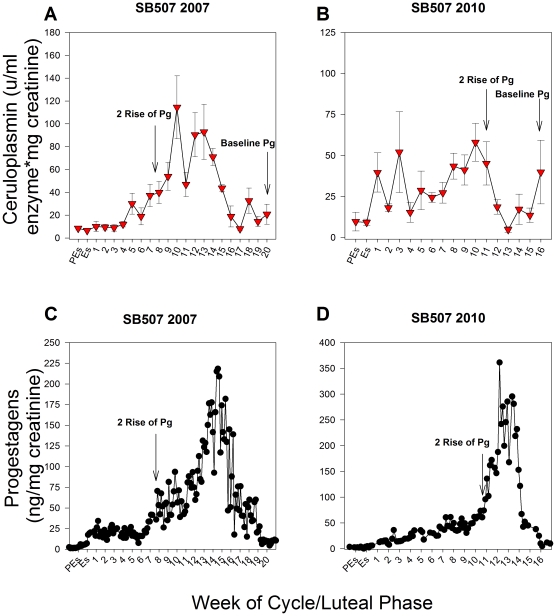
The levels of active urinary ceruloplasmin and urinary progestagens in lost pregnancies for female SB507. SB507 (MZS) was confirmed pregnant after artificial insemination in 2007 and 2010 by ultrasound at weeks 18 and 13, respectively; however, retarded growth was observed by ultrasound in the weeks thereafter and no birth occurred in either year. The levels of active urinary ceruloplasmin in 2007 (4A) and 2010 (4B) for SB507. Panels 4C and D: The levels of urinary progestagens in 2007 (4C) and 2010 (4D) for SB507. Red diamonds represent the levels active urinary ceruloplasmin; Black circles represent urinary progestagen concentrations; Pg = Progestagen; Pg = Progestagen; PEs = Proestrus; Es = Estrus; Numerical numbers = weeks of the luteal phase. Variations in the x and y axes exist to accommodate variable concentrations and durations. Data are weekly means ± SEM; n = 2 reproductive cycles.

Indications of pregnancy after AI were observed by ultrasound in animal SB507 (Memphis Zoological Society, MZS) in 2007 and 2010 but no birth occurred in either year. In 2007, a gestational sac was observed 2 weeks prior to the end of the luteal phase, or baseline progestagens. No further development was observed and the gestational sac degraded and regressed in the following weeks. In 2010, an early-stage fetus with a heartbeat was observed in the left uterine horn 3 weeks prior to baseline progestagens. However, no growth or heartbeat was observed the following week. The gestational sac then continued to degrade and regress in the weeks thereafter. In these confirmed lost pregnancies, the levels of active urinary ceruloplasmin were elevated during the luteal phase but the pattern was inconsistent with levels observed for term pregnancies (Figure 4A and B). For the luteal phase in 2007, levels of active ceruloplasmin did not increase until week 5 and a consistent increase above proestrus/estrus levels did not occur until week 7 (*P*≤0.05; Figure 4A). Interestingly, during the late luteal phase the levels of active urinary ceruloplasmin failed to remain at proestrus/estrus baseline values as was observed in pregnancies carried to term. After the initial decline during the late luteal phase, active ceruloplasmin increased again above baseline at week 18 (*P*≤0.05; Figure 4A). Active urinary ceruloplasmin increased by 5.4-fold above proestrus/estrus levels during the first week of the 2010 luteal phase, similar to what was observed in term pregnancies (*P*≤0.05). However, marked inconsistencies compared to term pregnancies were observed during the late luteal phase (Figure 4B). For example, the decline in ceruloplasmin during the end of the cycle occurred early in relation to the secondary rise in progestagens, with ceruloplasmin decreasing just 6 days after the secondary rise (Figure 4B and D). Thus, the levels of active ceruloplasmin remained elevated for only 14% of the secondary rise in progestagens compared to 42–71% in term pregnancies. In addition, although the levels of active urinary ceruloplasmin decreased to estrus/proestrus levels at week 12, they did not remain consistently low and increased above control levels at week 16 (*P*≤0.05), similar to the abnormal pattern observed during the late luteal phase of SB507's lost pregnancy in 2007 (Figure 4A).

**Figure 5:**
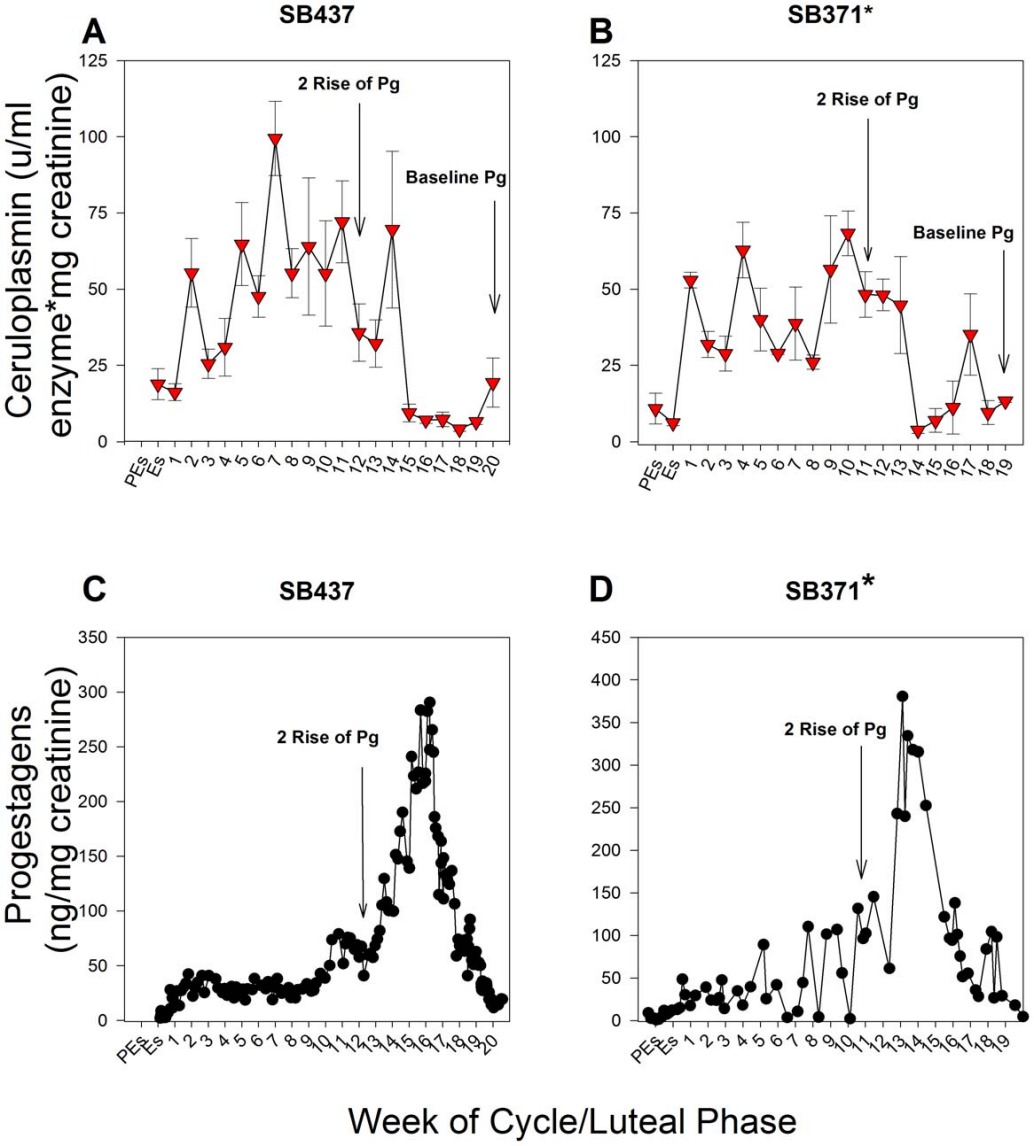
Representative patterns of active urinary ceruloplasmin and urinary progestagens in bred/no birth cycles for female giant pandas: suspected lost pregnancies based on ceruloplasmin. The levels of active urinary ceruloplasmin in 2008 for SB437 (SNZP; Panel 5A) and 2001 for SB371 (SDZ; Panel 5B). Both animals were artificially inseminated. Panels 5C and D: The levels of urinary progestagens for SB437 in 2008 (5C) and for SB371 in 2001 (4D). Red diamonds represent the levels active urinary ceruloplasmin; Black circles represent urinary progestagen concentrations; Pg = Progestagen; PEs = Proestrus; Es = Estrus; Numerical numbers = weeks of the luteal phase. Variations in the x axis exist to accommodate variable concentrations and durations. Data are weekly means ± SEM; n = 6 reproductive cycles, representative cycles (n = 2) are presented herein. *Samples from cycles obtained from SB371 were analyzed blind, without prior knowledge of cycle outcome. Because urinary progestagen concentrations for SB371 were analyzed using a different antibody, caution must be taken when directly comparing progestagen data between SB371 and the other animals. However, the same trends were observed in the relationship between active ceruloplasmin and progestagens in urine using both antibodies.

In the other female giant pandas examined, an increase in active urinary ceruloplasmin was observed for many cycles in which the animals were bred, primarily by AI, but no birth occurred. However, there were no ultrasound data to confirm these cycles as lost pregnancies, therefore we considered these cycles as suspected lost pregnancies based on ceruloplasmin. Similar to what was found in confirmed lost pregnancies, the pattern was different in these cycles compared to known term pregnancies (Figure 5A–B). While some of the suspected lost pregnancies were characterized as having a delayed increase in ceruloplasmin and/or a return of active ceruloplasmin to estrus/proestrus levels early in the luteal phase, all of the suspected lost pregnancies had deviations in the temporal pattern of urinary ceruloplasmin during the late luteal phase (*P*≤0.05). These deviations included an early decrease in active ceruloplasmin during the secondary rise in progestagens and/or increases in active ceruloplasmin after the initial decline during the late luteal phase, when compared to term pregnancies (Figure 5A–D). Out of the 7 total reproductive cycles in which animals were bred but no birth occurred, 6 cycles had elevated ceruloplasmin during the luteal phase (2 ultrasound-confirmed lost pregnancies and 4 suspected lost pregnancies based on ceruloplasmin).

### Active urinary ceruloplasmin in a suspected pseudopregnancy (no conception) after breeding or lost pregnancy that did not elicit an inflammatory response

**Figure 6:**
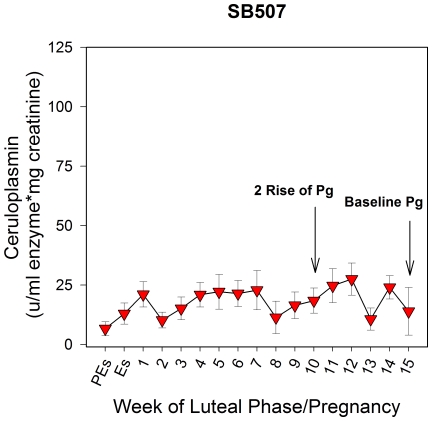
The levels of active ceruloplasmin in urine for a bred/no birth cycle for female SB507: suspected pseudopregnancy/no conception or lost pregnancy that did not elicit an immune response based on ceruloplasmin. In 2009, SB507 (MZS) was artificially inseminated but no change in the levels of active urinary ceruloplasmin was observed throughout the luteal phase and levels were similar to those observed at proestrus and estrus. Pg = Progestagen; PEs = Proestrus; Es = Estrus; Numerical numbers = weeks of the luteal phase. Data are weekly means ± SEM; n = 1 reproductive cycle.

In urine samples obtained from female SB507 (MZS) during the 2009 breeding season, no distinct increase in active ceruloplasmin was observed after AI (Figure 6). Urinary progestagens were monitored to ensure a normal luteal phase (data not shown) but the levels of active ceruloplasmin in urine remained consistent throughout the luteal phase, similar to the levels that were observed at proestrus and estrus (Figure 6); thus, the profile was most closely aligned to that of known pseudopregnant animals. Therefore, this cycle was considered a suspected pseudopregnancy/no conception or lost pregnancy that did not elicit an inflammatory response based on ceruloplasmin. This was the only cycle where a distinct increase in active urinary ceruloplasmin did not occur during the luteal phase out of the 7 total reproductive cycles included in this study where animals were bred naturally or by AI but birth did not take place.

## Discussion

Ex-situ conservation of the giant panda began in the 1960s. Due to problems like the lack of breeding in adults and high infant mortality, the giant panda's captive propagation history has been plagued by inconsistent reproductive success. In recent years, considerable improvement has been made in captive reproduction and cub survival due to the application of techniques like artificial insemination and improvement in husbandry and cub rearing [17]. The ex-situ population provides numerous benefits to the conservation of this species as a whole [1]. Opportunities for the advancement of basic science in understanding bear biology, a source of captive broodstock for reintroductions and translocations, and an assurance colony against wild population declines are a few of the reasons established by the International Union for the Conservation of Nature for maintaining a captive population [1]. After decades of research on the species' biology and ecology there still remain many unanswered questions, including those related to their reproductive strategy. Pseudopregnancy, delayed implantation/embryonic diapause, and the variable length of the luteal phase in both the pregnant and non-pregnant states are just some of the challenges that complicate research into the reproductive physiology of the giant panda. An accurate and straightforward pregnancy test for giant pandas has eluded scientists for decades, but the ability to distinguish between pregnancy and pseudopregnancy is indispensable for our basic understanding of this species' reproductive biology. The results described herein present the first physiological test to determine pregnancy status in this species through a non-invasive urinary assay that measures the acute phase protein ceruloplasmin.

Ceruloplasmin is part of a family of acute phase proteins that usually plays a protective role in response to an immune-provoking stimulus [18], [19]. It is a multifunctional copper containing protein that was first isolated in blood in 1948 [20]. One of its main roles is as an antioxidant, as it has substantial ferroxidase activity and can sequester other free radicals [18], [19], [21], [22]. Serum levels of ceruloplasmin have been found to increase during normal pregnancy in some species [14], [15] and it is thought that this increase during gestation protects against oxidative stress associated with pregnancy [18], [19], [21]. In dogs, the increase in serum ceruloplasmin has been coupled to the time of embryonic endometrial implantation and placentation [13], [16]. In contrast, our modified CEP assay showed that in giant pandas active ceruloplasmin in urine increases within one week after conception, indicating that this species produces an inflammatory response well before implantation of the embryo (Figure 2). Active urinary ceruloplasmin then remains elevated through approximately half of the secondary rise in progestagens (Figure 3), with the secondary rise being the presumed time of implantation. However, because the secondary rise in progestagens occurs in both pregnant and pseudopregnant animals, the exact timing of implantation remains unknown in giant pandas [2], [3], [5].

**Figure 7:**
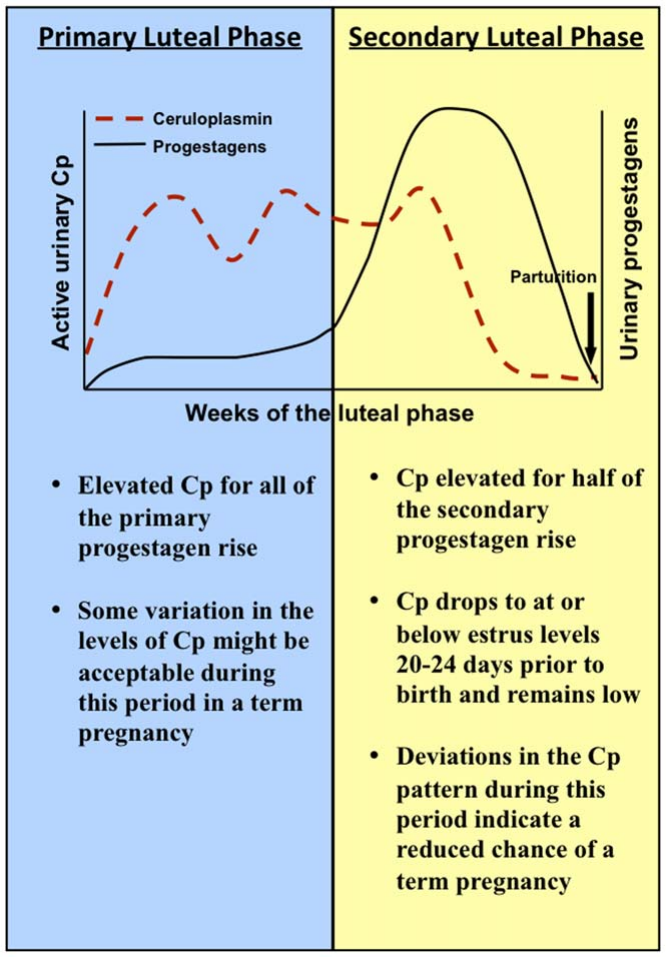
Schematic summary of elevated urinary ceruloplasmin and its relationship with progestagens during the pregnant luteal phase in the giant panda.

This study also suggests that the rate of pregnancy loss among captive giant pandas is quite high. In the two confirmed lost pregnancies and in suspected lost pregnancies based on elevated ceruloplasmin, the pattern deviated from the profile observed in pregnancies carried to term particularly in the latter part of the luteal phase (Figures 2–5). In term pregnancies, ceruloplasmin remained elevated until approximately half way through the secondary rise and then decreased late in the luteal phase to at or below control levels for the remainder of the cycle. In contrast, while some of the lost pregnancies showed more variability in the pattern of urinary ceruloplasmin early in the luteal phase, all of the confirmed and suspected lost pregnancies showed abnormalities in the pattern of ceruloplasmin during the late luteal phase, compared to the term pregnancy profiles. All of the cycles that had elevated ceruloplasmin without a birth showed a deviation in the temporal pattern of change toward the end of the luteal phase by reaching baseline close to the onset of the secondary rise in progestagens (the presumed time of embryo implantation) and/or did not remain low after the primary decline to at or below estrus/proestrus levels that occurred 20–24 days before birth in the term pregnancies. A schematic model of elevated ceruloplasmin and its relationship with progestagens during the pregnant luteal phase is depicted in Figure 7. Based on the results from this initial study, deviations in the pattern of urinary ceruloplasmin during the secondary luteal phase rise suggest a reduced chance of a term pregnancy. Analysis of additional term and confirmed lost pregnancies will help to elucidate the importance of the temporal pattern of change in relation to term pregnancies compared to lost pregnancies in the giant panda.

Interestingly, in SB507's (MZS) 2009 cycle, no significant increase was observed in the levels of active ceruloplasmin in urine during the luteal phase, indicating that this cycle may have been a pseudopregnancy when analyzing the cycle based on ceruloplasmin (Figure 6). However, it is also possible that this cycle was a lost pregnancy that did not elicit an appropriate inflammatory or immune response. In other species, there is evidence that immunological recognition is important for the maintenance of pregnancy and that an inappropriate maternal response to the fetus can result in fetal death [23], [24], [25], [26], [27], [28], [29]. A failed increase in, or an abnormal pattern of, active ceruloplasmin in urine following breeding or AI may be characteristic of an inappropriate response to pregnancy by the maternal immune system. Moreover, an unusual ceruloplasmin profile could also be a sign of abnormal embryonic development. No studies examining the specific temporal pattern of change in ceruloplasmin during pregnancy in relation to fetal loss have been carried out in other species. However, there is some evidence that either lower or higher levels of ceruloplasmin compared to levels observed during normal pregnancy could be an indication of problems associated with gestation in humans. Abnormal levels of ceruloplasmin have been found in blood and/or in amniotic fluid during pregnancies which resulted in such conditions as spontaneous abortions, preeclampsia, and Klinefelter's syndrome [30], [31], [32], [33], [34].

These data provide a foundation for understanding conception, fetal loss, and recognition and maintenance of pregnancy in giant pandas. More research will be needed to further verify the rate of lost pregnancies as well as to characterize the abnormal patterns in ceruloplasmin that we observed in known and suspected lost pregnancy cycles. Factors influencing pregnancy loss in captive animals will also need to be examined. If the rate of pregnancy loss is as high as this initial study suggests, ex-situ conservation efforts of this iconic endangered species will improve tremendously by subsequent investigations into the origin and mechanisms of pregnancy recognition and maintenance. Other ursids undergo both pseudopregnancy and delayed implantation of the embryo [35], [36], [37]. Thus, these studies will provide a background for future research in other endangered carnivores and ursids, such as the polar bear, in which common physiological factors are unreliable at determining pregnancy and the understanding of many components of basic reproductive biology is lacking.

## Materials and Methods

### Animals and sample collection

Urine from reproductive cycles of the four adult female giant pandas held at North American institutions was used for the study. Females SB507, SB437, SB452 and SB371 were housed at the Memphis Zoological Society (MZS), the Smithsonian National Zoological Park (SNZP), Zoo Atlanta (ZA) and the San Diego Zoo (SDZ), respectively. All animals were fed a diet consisting of at least 85–95% bamboo, with fruit and high fiber biscuits given as treats, and water was provided *ad libitum*. Urine samples were collected fresh, in a similar manner from each institution, via aspiration from the animal's enclosure and then stored frozen at −20°C. Sub-samples of urine (500 ul aliquots) from SNZP, ZA and SDZ were shipped frozen overnight to the MZS for ceruloplasmin analysis.

### Ceruloplasmin assay

Urine samples from sixteen estrous cycles (four cycles from each female) were used in the present study. Three to seven samples per week were analyzed for the weeks of proestrus (when available), estrus, and weeks 1 through the week of baseline progestagen levels or parturition of the luteal phase/pregnancy. Known pseudopregnant cycles when no breeding occurred were used as controls from each female.

Prior to being assayed for ceruloplasmin, urine samples were centrifuged at 3500 RPM for 10 minutes and concentrated to 300 µl. Samples were then assayed for ceruloplasmin through oxidasic activity measurement according to the optimizations of Sunderman and Nomoto, 1970 [38] with modifications. Briefly, the concentration of ceruloplasmin is determined by the rate of formation of a colored product from ceruloplasmin and the substrate, N,N-dimethyl-p-phenylenediamine (Sigma-Aldrich, St. Louis, MO). The rate of colorometric change is proportional to the amount of ceruloplasmin in each sample. The change in absorbance of the photometric product was measured every 30 seconds for a 5 minute interval using a Biomate 3 UV-Vis spectrophotometer (Thermo Scientific, Waltham, MA). The change in absorbance was also measured in a blank control for each run and this value was subtracted from the change in absorbance in each urine sample. Units of ceruloplasmin were calculated based on the difference in the change of absorbance in each sample from the control multiplied by reaction volume per the unit definition of change in absorbance at 550 nm (0.01), volume of enzyme used and the conversion factor for the published unit definition of a 7 ml reaction volume [39]. The inter-assay coefficient of variance was 14.9%. Creatinine was also measured in each urine sample to account for the concentration of water [3], [40], and final ceruloplasmin concentration was expressed per mg of creatinine. Ceruloplasmin assay validations are shown in the Materials and Methods S1, Figures S1 and S2 and Table S1.

### Progestagen enzyme immunoassays

An enzyme immunoassay (EIA) using the same progestagen antibody (CL425; C. Munro, UCDavis, CA) and conjugate (horseradish peroxidase progesterone conjugate; C. Munro, UCDavis, CA) was utilized to determine urinary progestagen concentrations for animals from SNZP, ZA and MZS. Urinary progestagen concentrations for SB371 (SDZ) were measured at SDZ using a single antibody EIA for the progesterone metabolite, pregnanediol–3-glucuronide (PdG; P-26; C. Munro, UCDavis, CA). Assay details are provided in the Materials and Methods S1. Both the group-specific CL425 progestagen antibody and the PdG specific antibody are widely used for captive management of the giant panda and a similar relationship was observed between active ceruloplasmin and the secondary rise of progestagens in urine for term pregnant cycles when concentrations of progestagens were analyzed using either antibody.

### Statistical analysis

Data sets for ceruloplasmin were tested for homogeneity of variance by Hartley's test [41], [42] and values were log transformed before statistical analysis, if heterogeneous variance was indicated. Changes in weekly ceruloplasmin levels for each cycle were analyzed using a one-way analysis of variance (ANOVA) using JMP 7 Statistical Discovery (SAS, Cary, NC). Dunnett's test was used to determine significant differences between weekly means of the luteal phase and that of estrus/proestrus (baseline control) level means. Means were considered different at *P*≤0.05. Graphed ceruloplasmin values are presented as weekly means ± SEM of non-transformed values and represent 3–7 urine samples/week.

For each luteal phase, the primary and secondary rise in urinary progestagens were determined by an iterative method as described previously for fecal progestagens [5]. Briefly, the primary rise was regarded as the time from the beginning of the luteal phase until the elevation of progestagens to 2 standard deviations above the mean for two or more days, while the secondary rise in urinary progestagens was the duration from the end of the primary rise until the end of the luteal phase. Termination of the luteal phase was defined as either parturition (term pregnancies) or the return to baseline progestagens (all other cycles).

## Supporting Information

Figure S1 **The effect of freeze-thaw cycles on the levels of active ceruloplasmin in urine obtained from giant pandas.** Repeated freeze-thaw cycles could affect the levels of active ceruloplasmin in urine. Ceruloplasmin activity was decreased by an average of 33, 42, 50, and 53% in the 2^nd^, 3^rd^, 4^th^, and 5^th^ thaws, respectively, compared to the activity observed at the initial measurement (set to 100%). Because banked samples had been frozen and thawed on numerous occasions and the exact number of freeze-thaw cycles was unknown and inconsistent between cycles, samples were not analyzed between different cycles. Data are the percent mean decrease in ceruloplasmin activity from the activity found at the initial thaw for the current study ± SEM; n = 3 samples.
(TIF)

Figure S2 **The effects of boiling on the levels of active ceruloplasmin in urine obtained from giant pandas.** Change in absorbance in each urine sample was measured prior to boiling and then after boiling for 10 or 30 minutes. Boiling for 10 minutes produced a decrease in activity by an average of 34%, while boiling for 30 minutes produced a decrease in activity by an average of 65%, compared to the ceruloplasmin activity observed with no treatment (set to 100%). Data are the percent mean decrease in ceruloplasmin activity from the activity found at the initial measurement ± SEM; n = 4 samples.
(TIF)

Table S1 **Comparison of active ceruloplasmin values in sample pools of giant panda urine obtained from the change in absorbance/ml enzyme method and standard curve method.** To check the validity that the rate of colorimetric change is proportional to the amount of ceruloplasmin in giant panda urine, values obtained from the change in absorbance/ml enzyme calculation method were compared to values obtained from a standard curve method. For the change in absorbance/ml enzyme calculation method, units of ceruloplasmin in two pools (a high and low pool) were calculated based on the difference in the change of absorbance in each sample from the blank control multiplied by reaction volume per unit definition for the change in absorbance at 550 nm (0.01), volume of enzyme used and the published conversion factor for the unit definition of a 7 ml reaction volume [39]. For the standard curve method, after subtracting the change of absorbance in each sample from the blank control, units of ceruloplasmin in each pool were calculated both manually using the linear regression (LR) equation obtained from the standard curve; y = 0.0067x−0.0011 and by using Sigma Plot software (Systat Software Inc., San Jose, CA) to calculate the values using a 4-parameter logistic (4PL) curve fit. A. The raw values of active ceruloplasmin for pool dilutions; values are expressed as u/ml enzyme* (change in absorbance/ml enzyme method) or u/ml* (standard curve method). B. Raw values of active ceruloplasmin by dilution factor; values are expressed as u/ml enzyme* (change in absorbance/ml enzyme method) or u/ml*(standard curve method). *Values are not expressed on a per mg of creatinine basis.
(DOC)

Materials and Methods S1 (DOC)
