# Supplementary figures and images for: The Acute Phase Protein Ceruloplasmin as a Non-Invasive Marker of Pseudopregnancy, Pregnancy, and Pregnancy Loss in the Giant Panda
Source: PLoS One. 2011 Jul 13;6(7):e21159. doi: 10.1371/journal.pone.0021159 (PMC3135589; doi:10.1371/journal.pone.0021159)

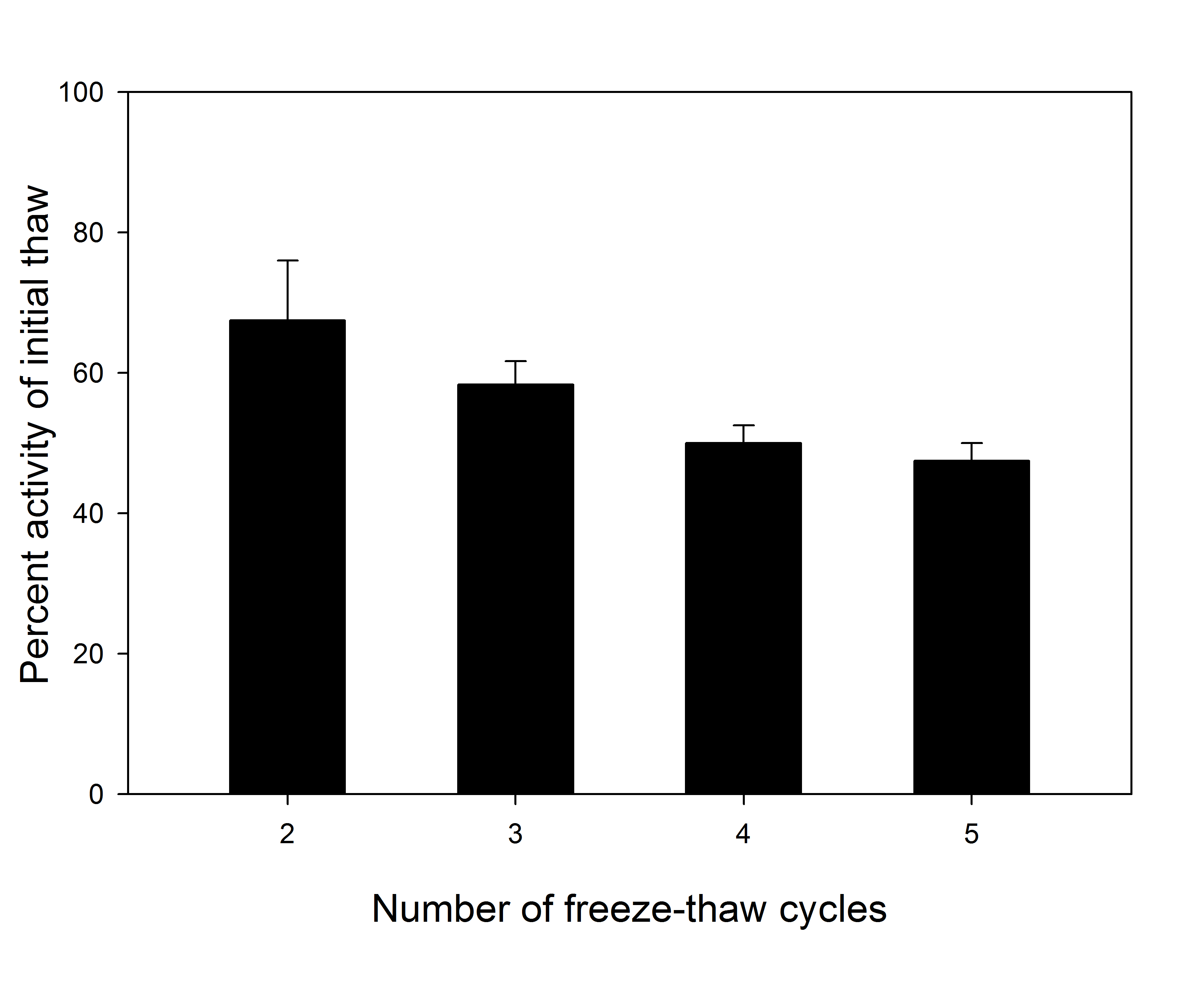

Supplement: Figure S1 — The effect of freeze-thaw cycles on the levels of active ceruloplasmin in urine obtained from giant pandas. Repeated freeze-thaw cycles could affect the levels of active ceruloplasmin in urine. Ceruloplasmin activity was decreased by an average of 33, 42, 50, and 53% in the 2nd, 3rd, 4th, and 5th thaws, respectively, compared to the activity observed at the initial measurement (set to 100%). Because banked samples had been frozen and thawed on numerous occasions and the exact number of freeze-thaw cycles was unknown and inconsistent between cycles, samples were not analyzed between different cycles. Data are the percent mean decrease in ceruloplasmin activity from the activity found at the initial thaw for the current study ± SEM; n = 3 samples. (TIF) [file pone.0021159.s001.tif]

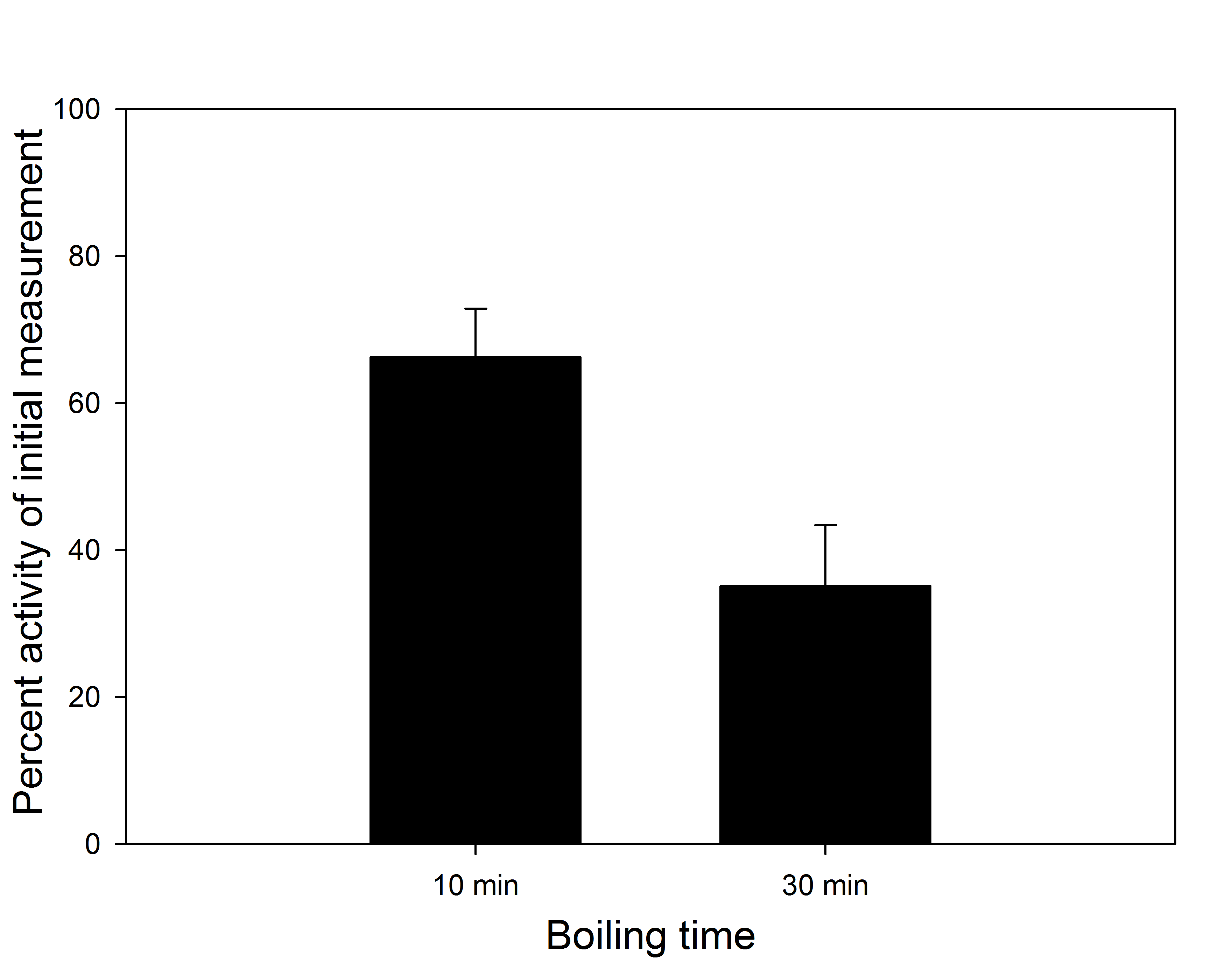

Supplement: Figure S2 — The effects of boiling on the levels of active ceruloplasmin in urine obtained from giant pandas. Change in absorbance in each urine sample was measured prior to boiling and then after boiling for 10 or 30 minutes. Boiling for 10 minutes produced a decrease in activity by an average of 34%, while boiling for 30 minutes produced a decrease in activity by an average of 65%, compared to the ceruloplasmin activity observed with no treatment (set to 100%). Data are the percent mean decrease in ceruloplasmin activity from the activity found at the initial measurement ± SEM; n = 4 samples. (TIF) [file pone.0021159.s002.tif]
