# Supplementary material for: The Acute Phase Protein Ceruloplasmin as a Non-Invasive Marker of Pseudopregnancy, Pregnancy, and Pregnancy Loss in the Giant Panda
Source: PLoS One. 2011 Jul 13;6(7):e21159. doi: 10.1371/journal.pone.0021159 (PMC3135589; doi:10.1371/journal.pone.0021159)
Supplement: Table S1 — Comparison of active ceruloplasmin values in sample pools of giant panda urine obtained from the change in absorbance/ml enzyme method and standard curve method. To check the validity that the rate of colorimetric change is proportional to the amount of ceruloplasmin in giant panda urine, values obtained from the change in absorbance/ml enzyme calculation method were compared to values obtained from a standard curve method. For the change in absorbance/ml enzyme calculation method, units of ceruloplasmin in two pools (a high and low pool) were calculated based on the difference in the change of absorbance in each sample from the blank control multiplied by reaction volume per unit definition for the change in absorbance at 550 nm (0.01), volume of enzyme used and the published conversion factor for the unit definition of a 7 ml reaction volume [39]. For the standard curve method, after subtracting the change of absorbance in each sample from the blank control, units of ceruloplasmin in each pool were calculated both manually using the linear regression (LR) equation obtained from the standard curve; y = 0.0067x−0.0011 and by using Sigma Plot software (Systat Software Inc., San Jose, CA) to calculate the values using a 4-parameter logistic (4PL) curve fit. A. The raw values of active ceruloplasmin for pool dilutions; values are expressed as u/ml enzyme* (change in absorbance/ml enzyme method) or u/ml* (standard curve method). B. Raw values of active ceruloplasmin by dilution factor; values are expressed as u/ml enzyme* (change in absorbance/ml enzyme method) or u/ml*(standard curve method). *Values are not expressed on a per mg of creatinine basis. (DOC) [file pone.0021159.s003.doc]

|  |  |  | **A. Raw Values** | | **B. By Dilution Factor** | | |
| --- | --- | --- | --- | --- | --- | --- | --- |
|  |  | **ml Enzyme** | **Std Curve-LR** | **Std Curve-4PL** | **ml Enzyme** | **Std Curve-LR** | **Std Curve-4PL** |
|  | Dilution | u/ml enzyme* | u/ml* | u/ml* | u/ml enzyme* | u/ml* | u/ml* |
| **Pool 1** | Neat | 1.68 | 1.36 | 1.44 | 1.68 | 1.36 | 1.44 |
| **(low)** | 1:2 | 1.05 | 0.91 | 0.96 | 2.10 | 1.82 | 1.93 |
|  | 1:4 | 0.63 | 0.61 | 0.63 | 2.52 | 2.45 | 2.52 |
|  | 1:8 | 0.21 | 0.31 | 0.27 | 1.68 | 2.51 | 2.13 |
| **Pool 2** | Neat | 8.19 | 5.99 | 5.94 | 8.19 | 5.99 | 5.94 |
| **(high)** | 1:2 | 5.25 | 3.90 | 3.93 | 10.50 | 7.79 | 7.87 |
|  | 1:4 | 3.57 | 2.70 | 2.78 | 14.28 | 10.81 | 11.12 |
|  | 1:8 | 2.10 | 1.66 | 1.74 | 16.80 | 13.25 | 13.96 |
