# Supplementary material for: The Acute Phase Protein Ceruloplasmin as a Non-Invasive Marker of Pseudopregnancy, Pregnancy, and Pregnancy Loss in the Giant Panda
Source: PLoS One. 2011 Jul 13;6(7):e21159. doi: 10.1371/journal.pone.0021159 (PMC3135589; doi:10.1371/journal.pone.0021159)
Supplement: Materials and Methods S1 — (DOC) [file pone.0021159.s004.doc]

**Materials and Methods S1**

**Verification of a protein similar in molecular weight to human ceruloplasmin in giant panda urine by Matrix Assisted Laser Desorption/Ionization (MALDI) mass spectrometry**

MALDI mass spectrometry was performed at Mississippi State University. Giant panda urine from 3 samples with high ceruloplasmin activity, one sample with extremely low ceruloplasmin activity (negative control) and a purified human ceruloplasmin control (Sigma-Aldrich, St. Louis, MO) were prepared using the dried-droplet method, as previously described [43]. MADLI analysis was performed using a Bruker Microflex MALDI time of flight mass spectrometer (Bruker Daltonic, Billerica, MA). Only the high mass region was scanned and once a peak was indentified for the human ceruloplasmin control, peaks with similar molecular weights were focused on in the urine samples, and their mass to charge (m/z) values were determined. A protein with similar characteristics and molecular weight to human ceruloplasmin was verified in giant panda urine known to have high levels of ceruloplasmin activity; a peak was observed at 138930.55 m/z, corresponding to ~139 kDa in giant panda urine compared to a peak at 134920.11 m/z, corresponding to a molecular weight ~135 kDa in the human ceruloplasmin control. Ceruloplasmin heterogeneity has previously been found between species (as well as within species due to multiple splice variants, etc.) [44,45,46,47,48]. No defined peaks within this mass range and a very large signal to noise ratio were observed in the negative control urine sample (data not shown).

**Progestagen enzyme immunoassays**

An enzyme immunoassay (EIA) using the same progestagen antibody (CL425; C. Munro, UCDavis, CA) and conjugate (horseradish peroxidase progesterone conjugate; C. Munro, UCDavis, CA) was utilized to determine urinary progestagen concentrations for animals from SNZP, ZA and MZS. However, different institutions performed the hormone analyses. For SB437 (SNZP) and SB452 (ZA), urinary progestagens were measured at the SNZP as previously described [5,6,49]. The concentration of progestagens in urine for SB507 (MZS) was measured at the MZS by a similar single antibody EIA. Urinary progestagens were measured using the broad scale [49] progesterone antibody (CL425) and horseradish peroxidase progesterone conjugate provided by C. Munro (UCDavis, CA) and a commercially available progesterone was used for standards (4-Pregnene-3,20-dione; Sigma-Aldrich, St. Louis, MO). The antibody was used at a final dilution of 1:6,000 and the sensitivity of the assay was 0.01 ng/ml. Samples were diluted in 0.1 M phosphate buffered saline (19.5% 0.2 M monobasic sodium phosphate, 30.5% 0.2 M dibasic sodium phosphate , 8.7% NaCl, and 1% bovine serum albumin; Sigma-Aldrich, St. Louis, MO) prior to being assayed. Standards, samples, and controls were added (50 µl/well) to antibody-coated microtiter plates. Progesterone conjugate at a final dilution of 1:60,000 was added to each well (50 µl/well) and incubated for two hours. After incubation, unbound hormone was removed and the substrate solution (10 % 2,2′-Azino-bis(3-ethylbenzothiazoline-6-sulfonic acid) diammonium salt and 3.2% hydrogen peroxide in 9.6 % citric acid; Sigma-Aldrich, St. Louis, MO) was added (100 µl/well) and allowed to develop in the dark until optical density had reached 1 in the maximum binding wells (~45-60 minutes). Optical densities were read with a Dynex Technologies MRX microtiter plate reader **(**Thermo Labsystems, Chantilly, VA). The intra- and inter-assay coefficients of variance were 3.0 % and 10.2 % and 5.4 % and 14.5 %, respectively for the low and high controls. Creatinine was measured in each urine sample to account for the concentration of water [3,50] and progestagen concentration was expressed as mg of creatinine.

Urinary progestagen concentrations for SB371 (SDZ) were measured at SDZ using a single antibody EIA for the progesterone metabolite, pregnanediol-3-glucuronide, as previously described [51,52,53]. In this assay, PdG antiserum (P-26; C. Munro, UCDavis, CA) is combined with horseradish peroxidase PdG conjugate (C. Munro, UCDavis, CA). Both the group-specific CL425 progestagen antibody and the PdG specific antibody are widely used for captive management of the giant panda and a similar relationship was observed between active ceruloplasmin and the secondary rise of progestagens in urine for term pregnant cycles when concentrations of progestagens were analyzed using either antibody.
